# Intramuscular Pressure of Tibialis Anterior Reflects Ankle Torque but Does Not Follow Joint Angle-Torque Relationship

**DOI:** 10.3389/fphys.2018.00022

**Published:** 2018-01-24

**Authors:** Filiz Ateş, Brenda L. Davies, Swati Chopra, Krista Coleman-Wood, William J. Litchy, Kenton R. Kaufman

**Affiliations:** ^1^Motion Analysis Laboratory, Department of Orthopedic Surgery, Mayo Clinic, Rochester, MN, United States; ^2^Department of Neurology, Mayo Clinic, Rochester, MN, United States

**Keywords:** intramuscular pressure, electromyography, surface EMG, fine-wire EMG, tibialis anterior, force prediction, electromechanical delay

## Abstract

Intramuscular pressure (IMP) is the hydrostatic fluid pressure that is directly related to muscle force production. Electromechanical delay (EMD) provides a link between mechanical and electrophysiological quantities and IMP has potential to detect local electromechanical changes. The goal of this study was to assess the relationship of IMP with the mechanical and electrical characteristics of the tibialis anterior muscle (TA) activity at different ankle positions. We hypothesized that (1) the TA IMP and the surface EMG (sEMG) and fine-wire EMG (fwEMG) correlate to ankle joint torque, (2) the isometric force of TA increases at increased muscle lengths, which were imposed by a change in ankle angle and IMP follows the length-tension relationship characteristics, and (3) the electromechanical delay (EMD) is greater than the EMD of IMP during isometric contractions. Fourteen healthy adults [7 female; mean (*SD*) age = 26.9 (4.2) years old with 25.9 (5.5) kg/m^2^ body mass index] performed (i) three isometric dorsiflexion (DF) maximum voluntary contraction (MVC) and (ii) three isometric DF ramp contractions from 0 to 80% MVC at rate of 15% MVC/second at DF, Neutral, and plantarflexion (PF) positions. Ankle torque, IMP, TA fwEMG, and TA sEMG were measured simultaneously. The IMP, fwEMG, and sEMG were significantly correlated to the ankle torque during ramp contractions at each ankle position tested. This suggests that IMP captures *in vivo* mechanical properties of active muscles. The ankle torque changed significantly at different ankle positions however, the IMP did not reflect the change. This is explained with the opposing effects of higher compartmental pressure at DF in contrast to the increased force at PF position. Additionally, the onset of IMP activity is found to be significantly earlier than the onset of force which indicates that IMP can be designed to detect muscular changes in the course of neuromuscular diseases impairing electromechanical transmission.

## Introduction

As muscles play a key role in movement, one of our research goals is to detect changes in the musculature due to neuromuscular diseases, exercise, or aging by relating an individual muscle's function, i.e., the ability to exert force, with its effects on the joint(s) it crosses. However, direct *in vivo* measurements of human muscle forces necessitates intraoperative approaches (e.g., Ates et al., 2016). Such approaches are, however, very rare. This is not only due to the fact that such measurements are hard to perform but also due to the fact that intraoperative measurements preclude voluntary muscle activities. Ultrasound imaging can be used to quantify forces from e.g., Achilles tendon (Dick et al., 2016) or fiber optic sensors measure volitional forces directly from tendons (Komi et al., 1996). However, they do not provide information on individual muscle forces. The fiber optic measurements have also reported to be susceptible to high errors due to skin interactions (Erdemir et al., 2003). Therefore, characterization of individual muscles' contribution to joint mechanics is necessary either by indirect, non-invasive approaches, e.g., shear wave elastography (Nordez and Hug, 2010; Hug et al., 2015) based on a linear relationship between muscle stiffness and active forces (Ateş et al., 2015), and electromyography (EMG), or by direct, minimally invasive methods, e.g., intramuscular pressure (IMP) measurements.

IMP is defined as the hydrostatic fluid pressure within a muscle. Earlier studies suggested that IMP represents muscle tension (Baumann et al., 1979) since the pressure applied by muscle fibers on interstitial fluid is directly related to muscle force production. IMP has been measured in a number of muscles and subject to different conditions, e.g., the vastus medialis during isometric contractions (Sejersted et al., 1984), the vastus lateralis during isokinetic contractions (Crenshaw et al., 2000), as well as the tibialis anterior (TA) and the soleus muscles during eccentric and concentric contractions (Aratow et al., 1993). In general, IMP studies provide promising findings for many clinical applications and conditions affecting muscular pressure like compartment syndrome (Sejersted and Hargens, 1995). More recently, a minimally invasive pressure microsensor has been developed to measure IMP (Davis et al., 2003; Kaufman et al., 2003). In this case, a fiber optic sensor measures the pressure through a diaphragm that deforms due to alterations in fluid pressure and subsequently changes the output signal. This advancement improves the functionality of the minimally invasive method and provides accurate quantification of interstitial fluid pressure. However, *in situ* animal experiments performed on isolated muscles suffer from not representing intact behavior of musculature (Davis et al., 2003). In order to detect muscular changes in relation to joint position, it is essential to carry out a direct comparison of *in vivo* muscle activity and IMP measurements.

While IMP sensor measures pressure active and passive mechanical characteristics of muscle, EMG is capable of quantifying the neuromuscular electrical activity of individual muscles. Although, it is known that EMG does not provide a quantitative measurement of muscle force, it has been successfully used to estimate muscular forces (Perry and Bekey, 1981; Solomonow et al., 1990). Collecting the resulting potential fields of a contracting muscle with a significantly sized surface electrode, i.e., measuring surface EMG (sEMG) can provide activation information for superficial muscles only. Fine-wire EMG (fwEMG), on the other hand, provides local contraction information from deeper muscles. Further, crosstalk, one of the main challenges of sEMG, has been reported to play a minor role for most of the biomechanical applications involving fwEMG (Solomonow et al., 1994). While there exist many studies that compare IMP information with sEMG (Aratow et al., 1993; Crenshaw et al., 2000; Davis et al., 2003), comparing IMP with fwEMG signals from the vicinity of an IMP sensor has not yet been studied.

Electromechanical delay (EMD) is due to the natural link between mechanical and electrophysiological muscle characteristics. EMD is defined as the time lag between the activation of a muscle and force production at the tendon due to the underlying electromechanical processes of excitation-contraction coupling (Norman and Komi, 1979; Le Mansec et al., 2017). EMD provides a link between mechanical and electrophysiological quantities (Nordez et al., 2009). Pathological conditions such as dystrophy (Lacourpaille et al., 2017) or spasticity (Granata et al., 2000) influence the EMD. Therefore, it is critical to detect changes in EMD due to muscle diseases such as myasthenia gravis or Lambert-Eaton myasthenic syndrome where the electromechanical coupling is disrupted (Chiou-Tan and Gilchrist, 2015).

The goal of this study was to assess the relationship of IMP with respect to the mechanical and electrical characteristics of the TA muscle activity at different ankle joint positions. We hypothesized that (1) the IMP of TA and the sEMG and fwEMG correlate to the ankle joint torque, (2) the isometric force of TA increases at increased muscle lengths, which were imposed by a change in ankle angle and IMP follows the muscle length-tension characteristics (3) the EMD between the ankle torque and the fwEMG is greater than the onset delay of IMP with respect to fwEMG.

## Methods

### Participants

Fourteen young healthy adults between the ages of 21–40 years old [7 females, 7 males; mean (*SD*) = 26.9 (4.2) years old with 25.9 (5.5) kg/m^2^ body mass index] participated. The exclusion criteria were (i) history of nervous system disorders or musculoskeletal diseases: musculoskeletal pathology, myopathy, (ii) history of surgeries on the right lower extremity, (iii) current use of blood thinners or medications that manipulate muscle strength like corticosteroids or statins, and (iv) pregnancy for females. The procedures were approved by the Mayo Clinic Institutional Review Board (IRB). All participants gave written informed consent.

### Experiments

**Figure 1:**
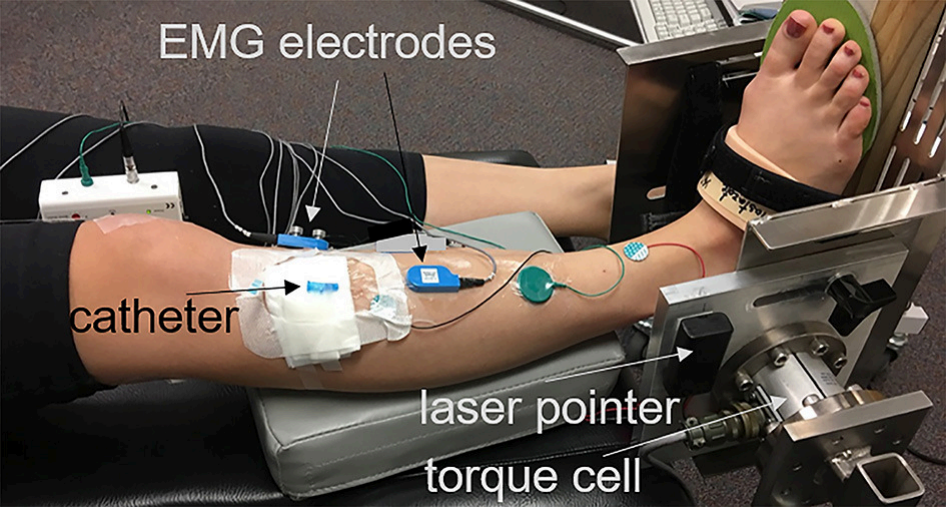
Right foot of the participant strapped into a torque measurement system. The center of the torque cell aligned to the axis of the ankle with the help of a laser pointer whose light got in line alongside the lateral malleolus. A wooden wedge with an angle of 20° placed underneath the foot in order to promote exclusive activation of the TA muscle and optimal line of action.

The participant was positioned supine with the foot secured to a custom torque measurement system (Figure 1): A torque cell (Honeywell, Model 2110-5K; Honeywell International Inc., Morris Plains, NJ, USA. Non-linearity: ±0.1% of rated output. Hysteresis: ±0.1% of rated output. Repeatability: ±0.05% of rated output) with a max output of 564.9 N was attached to an aluminum device designed to fix the ankle angle. Test-retest reliability of the torque measurement system was confirmed: Intraclass correlation coefficient equaled 0.88 and 0.96 for plantarflexion and dorsiflexion maximum voluntary contraction (MVC). The torque cell, connected to a strain gage amplifier (SGA/A, Mantracourt Electronics Ltd. Exeter, UK), was calibrated with known weights prior to experiments.

The center of the torque cell was aligned with the axis of the right ankle of the participant. This was done with the help of a laser pointer whose light was aligned with the lateral malleolus. A wooden wedge with an angle of 20° was placed underneath the foot (Figure 1). Since the TA also assists in inversion of the foot, this ankle position ensured that the line of action of the TA muscle was aligned with the torque measurement system and optimized its contribution to the measured joint torque. This position also helped minimize the contribution of other dorsiflexor muscles; e.g., extensor digitorum longus (EDL) and peroneus tertius, that are responsible from eversion of the foot (Kendall et al., 1993). Tests were performed with the ankle immobilized (i) at dorsiflexion position (DF) between 5° and 10°, (ii) in neutral position (i.e., 90°) (Neutral), and (iii) at 20° plantar flexion position (PF).

The skin over the TA was shaved and cleaned. The motor end plate localization in the TA is well-established (Aquilonius et al., 1984). The location of motor unit end plates was verified with percutaneous stimulation of the peroneal nerve and recording the compound muscle action potential (CMAP) using a Nicolet Viking EDX instrument (Natus Neurology, Madison, WI). The location of the active recording surface electrode was marked for future reference. For IMP measurements, a 22-gauge IV catheter (Introcan Safety, B. Braun, Medical Inc., Bethlehem, PA) was inserted parallel to the muscle fibers and positioned between the deep surface of the crural fascia and the central tendon using ultrasound guidance (ACUSON Freestyle, Siemens Medical Solutions USA, Inc., Mountain View, CA). The tip of the needle was inserted ~2 cm proximal to the region of the motor end plates. After stylet of the catheter was removed, a fiber optic pressure sensor (FOP-M260, FISO Technologies, Inc., Quebec, Canada) was inserted. The tip of the sensor was positioned 1 cm beyond the tip of the catheter lumen. The fiber optic sensor was attached to the instrumentation system (FPI-LS-10 Module on EVO-SD-5 Evolution Chassis, FISO Technologies, Inc., Quebec, Canada) configured for a 3000 Hz sampling frequency. A fine-wire EMG electrode was inserted in close proximity to the pressure sensor, verified by ultrasound, and connected to an EMG preamplifier (Y03, Motion Lab Systems, Baton Rouge, LA). The location of fwEMG electrode was around 1 cm proximal to the motor end plate region. The catheter was supported with gauze secured with adhesive tape and the area was covered with Tegaderm (3M, St. Paul, MN, USA). Surface EMG electrodes with an EMG preamplifier (Z03, Motion Lab Systems, Baton Rouge, LA) were placed on the skin near the motor end plate region. A ground electrode was placed on the subject's ankle. The EMG signals were collected using the MA300-XVI data collection hardware configured with a 10 Hz high-pass and 2000 Hz low-pass filter (Motion Lab Systems, Baton Rouge, LA).

The participants performed (i) three isometric dorsiflexion MVC for 4 s and (ii) three isometric ramp contractions from 0% to 80% MVC at rate of 15% MVC/second at each ankle position. The ankle torque, sEMG, fwEMG, and IMP signals were simultaneously acquired and digitized at 3000 Hz with a 16-bit analog to digital converter (NI USB-6225, National Instruments, Austin, TX, USA) using custom software (LabVIEW, National Instruments, Austin, TX, USA). Visual feedback was provided to the participant by the same custom software during the experiments. To prevent muscle fatigue, a minimum 30 s rest was provided between trials.

### Data analysis

Data processing was performed using custom MATLAB software (The MathWorks, Natick, MA). Raw IMP and torque signals were filtered with a dual-pass 50 Hz 4th-order low pass Butterworth filter. Raw fw- and sEMG signals were full-wave rectified and sEMG was filtered with a dual-pass 10–500 Hz 4th-order band pass Butterworth filter whereas fwEMG was filtered with a dual-pass 10–1500 Hz 4th-order band pass Butterworth filter to remove baseline noise and minimize signal artifacts (De Luca et al., 2010). The root-mean-square (RMS) of the EMG data was calculated using a 250-point moving average window.

#### MVC data

Average peak ankle torque values were calculated from the mid-2 s of each MVC trial. Three trials were averaged for each participant and the average of the torques of all participants was calculated for each position tested. RMS values for fw- and sEMG of TA during MVC trials were calculated and used to normalize EMG signals collected during isometric ramp contractions.

#### Isometric ramp contractions

RMS values for fwEMG and sEMG of TA were calculated for each trial and normalized to the peak RMS values calculated from MVC data of each ankle position.

For all ramp data, three trials were averaged point by point for each participant and averages of all participants and standard deviations were calculated for each position tested.

To test the electromechanical delay (EMD), the average and standard deviation of the quiescent signal (the initial 500 ms of each data collection) was calculated. The onset of signals was defined as the time when the signal was greater than three standard deviations above the average quiescent value for at least 25 ms (Di Fabio, 1987). The onsets were identified using a custom algorithm (MATLAB) and confirmed visually for each tracing within each trial. The delay between the onset of ankle torque and IMP with respect to the fwEMG onset was quantified for each trial as EMD. Differences between the onset of fw- and sEMG were calculated as well.

### Statistical analyses

Custom MATLAB software (The MathWorks, Natick, MA) was used for statistical analyses. Distribution normality was tested using the Kolmogorov–Smirnov test and statistical tests were selected accordingly. The effects of ankle position on ankle torque, IMP, fw-, and sEMG RMS of TA during MVC were tested using repeated measures ANOVA with statistical significance set at *p* = 0.05. *Post-hoc* tests with Bonferroni correction was used to detect further differences. The Spearman's rank correlation coefficient was calculated to analyze the relationship between IMP, fwEMG RMS, sEMG RMS, and ankle torque during ramp contractions at each position. Additionally, the Spearman's rank correlation coefficient was calculated by pooling peak IMP and torque values for all joint positions to analyze if the relationship is consistent across the joint angles. A Kruskal–Wallis test was performed to detect differences between the onset delays of IMP, ankle torque, and sEMG with respect to fwEMG.

## Results

### MVC

**Figure 2:**
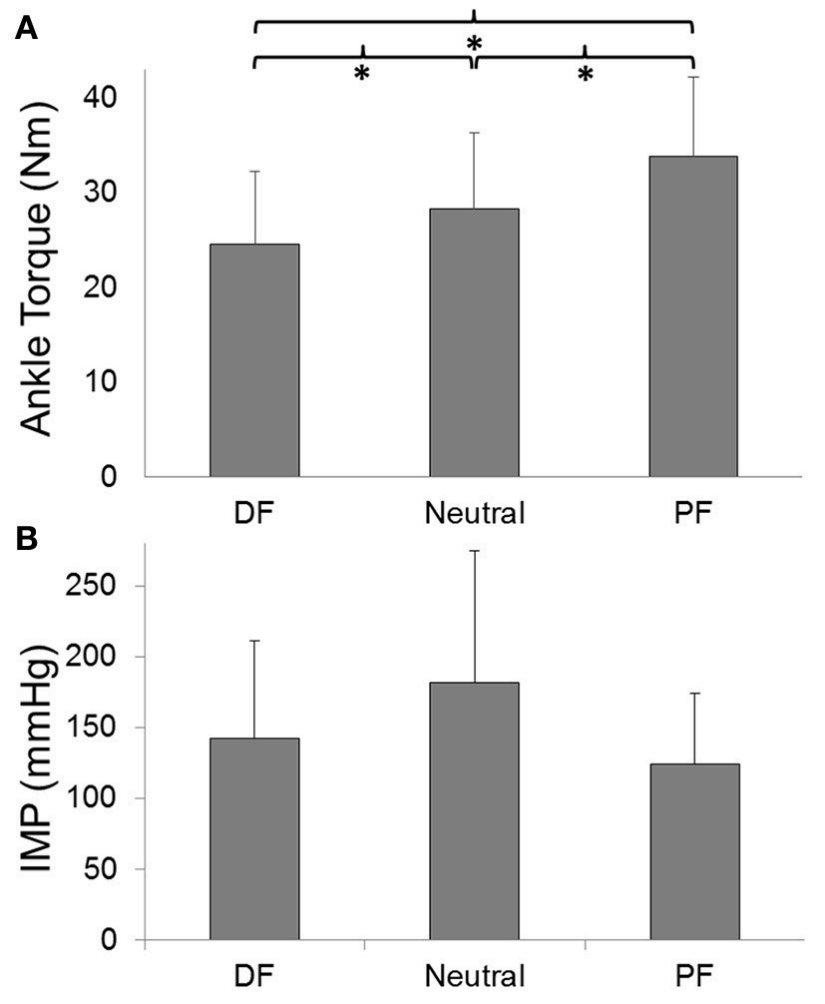
**(A)** TA peak forces and **(B)** IMP responses during maximum voluntary contraction (MVC) with respect to ankle angle. ^*^Indicates significant difference.

The ankle torque changed significantly at different ankle positions (*p* < 0.001) (Figure 2A). *Post-hoc* tests showed that the ankle torque produced at PF [mean (*SD*) = 33.8 (8.4) Nm] was significantly higher than the torque produced in Neutral [28.3 (8.0) Nm; *p* = 0.003] and DF [24.5 (7.7) N; *p* < 0.001] by 19.6% and 38.1%, respectively. Peak torque produced in Neutral was significantly higher than the torque produced at DF (by 15.5%; *p* = 0.002). The changes in IMP (*p* = 0.1; Figure 2B) as well as the RMS of fwEMG (*p* = 0.2) and sEMG (*p* = 0.2) of the TA during MVC were not significant with respect to the changes in ankle positions.

### Isometric ramp contractions

**Figure 3:**
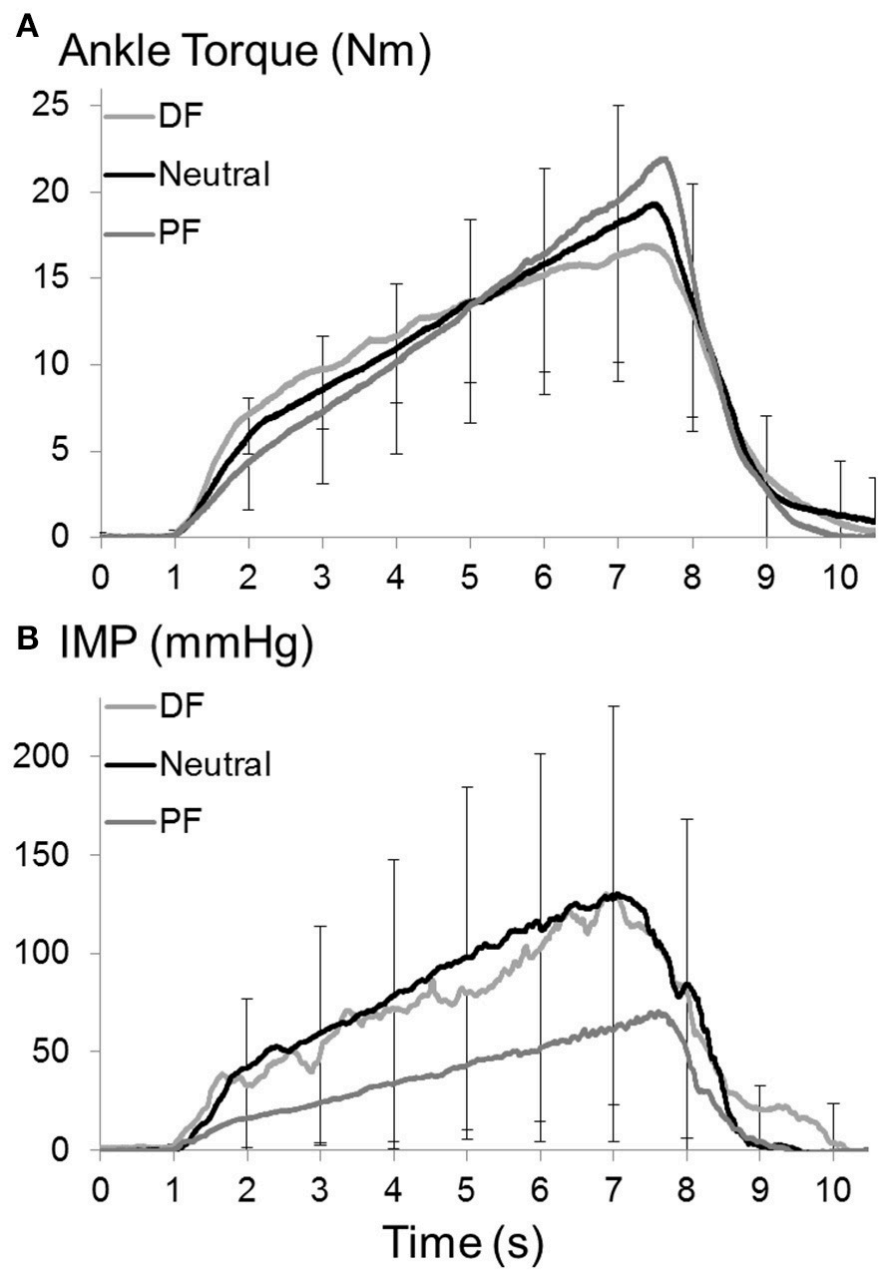
**(A)** Ankle torque and **(B)** IMP of TA muscle during isometric ramp contractions up to 80% MVC at dorsiflexion (DF), neutral (Neutral), and plantarflexion (PF) positions of ankle. Raw IMP and force signals were filtered with a dual-pass 50 Hz 4th-order low pass Butterworth filter. Three ramp contraction trials of each participant were averaged point by point and averages of all participants were calculated and plotted for each position. Error bars set one for 3,000 points (per second) show standard deviation.

**Figure 4:**
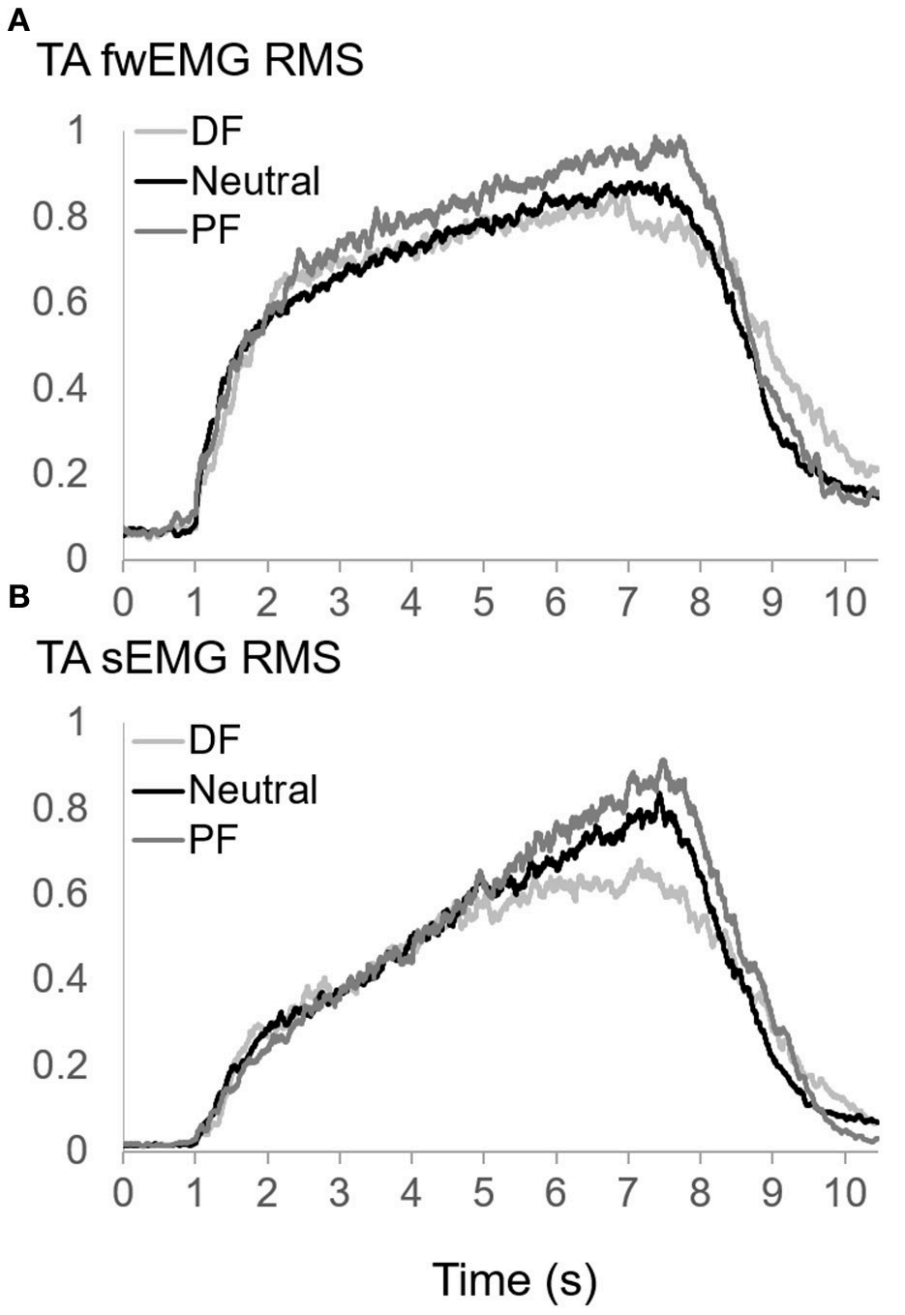
**(A)** Fine-wire EMG (fwEMG) root-mean-square (RMS), and **(B)** surface EMG (sEMG) RMS of TA during isometric ramp contractions up to 80% MVC at dorsiflexion (DF), neutral (Neutral), and plantarflexion (PF) positions of ankle. Raw fw- and sEMG signals were full-wave rectified. sEMG was filtered with a dual-pass 10–500 Hz 4th-order band pass Butterworth filter whereas fwEMG was filtered with a dual-pass 10–1500 Hz 4th-order band pass Butterworth filter. EMG RMS was calculated using a 250-point moving average window. RMS values of each trial normalized to the peak RMS values calculated from MVC values at each position. Three trials of each participant were averaged point by point and averages of all participants were calculated and plotted for each position.

The IMP (Figure 3B), fwEMG (Figure 4A), and sEMG (Figure 4B) showed significant correlations with the ankle torque (Figure 3A) at every ankle positions tested (Table 1). However, IMP and torque were not significantly correlated (ρ = 0.14, *p* = 0.45) when the values at different ankle joint angles were pooled and analyzed together.

**Table 1:** Spearman correlation coefficients between TA muscle pressure, EMG RMS, and ankle torque at different ankle positions.

**Correlation**	**DF (ρ)**	**Neutral (ρ)**	**PF (ρ)**
IMP vs. RMS fwEMG	0.72	0.73	0.69
IMP vs. RMS sEMG	0.71	0.70	0.72
IMP vs. Torque	0.90	0.95	0.95
RMS fwEMG vs. RMS sEMG	0.86	0.83	0.83
RMS sEMG vs. Torque	0.92	0.96	0.97
RMS fwEMG vs. Torque	0.84	0.86	0.89

*All correlations are statistically significant*.

**Figure 5:**
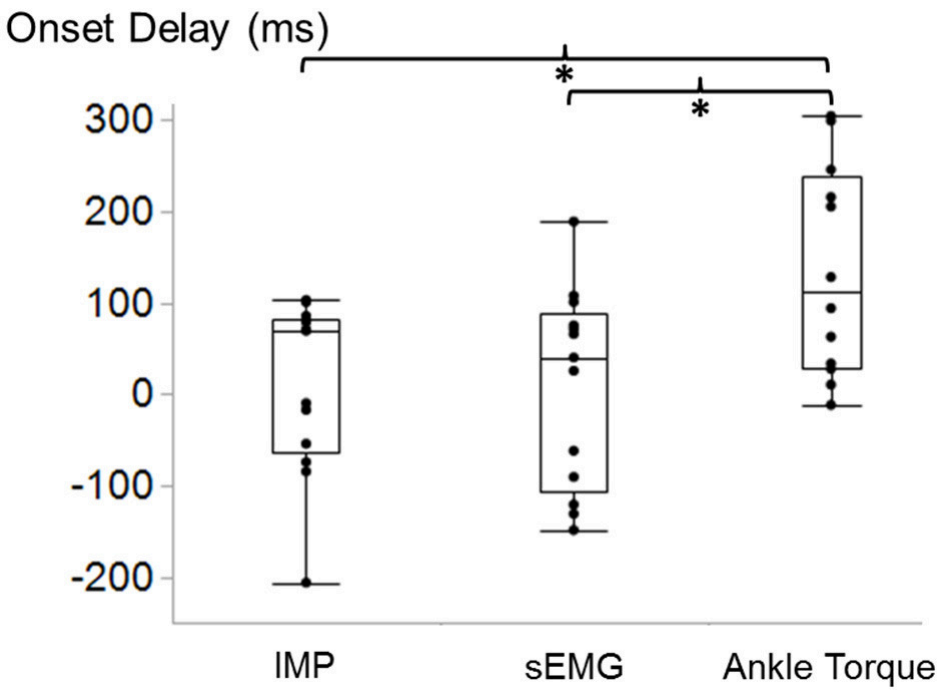
Box and whisker plot of TA IMP, surface EMG (sEMG), and ankle torque electromechanical delay (EMD) with respect to the onset time of fine-wire EMG calculated during ramp contractions. ^*^Indicates significant difference.

Ankle position did not have any significant effects on onset delays of IMP (*p* = 0.9), sEMG (*p* = 0.3), and torque (*p* = 0.8) with respect to fwEMG. The average EMD measured at three positions for IMP, ankle torque, and sEMG was significantly different from each other (*p* = 0.02). The ankle torque EMD [median (interquartile range) = 111.2 (191.2) ms] was significantly greater than the onset delay of IMP [69.5 (134.9) ms; *p* = 0.02]. Additionally, the torque EMD was significantly greater than the difference between the onset of sEMG and fwEMG (40.2 (165.9) ms) (*p* = 0.03) (Figure 5).

## Discussion

The results demonstrate significant correlations between ankle torque, IMP, sEMG, and fwEMG during isometric ramp contractions within the ankle joints tested. Even though, the change in torque values imposed by a change in ankle position was not reflected as a change in the peak IMP, the IMP follows the ramp contractions at each trial at each position. This confirms our first hypothesis. It was recently shown that the relationship between a TA muscle's IMP and force did not change with the rate of force increase of 5, 10, and 15% MVC per second (Go et al., 2016). This is also consistent with the previous report (Lawrence and De Luca, 1983) showing that force-EMG relationship was independent of the rate of force increase (10, 20, and 40% MVC per second) for biceps, deltoid, and first dorsal interosseous muscles. Therefore, the choice of a rate of 15% MVC is reasonable, as one is able to reach high force values in reasonable time.

Within this work, it was assumed that most of the ankle torque is provided by the TA. It is not possible to fully eliminate the contribution of other muscles to ankle dorsiflexion torque measurements however; the inversion foot positioning during dorsiflexion does optimize TA impact while simultaneously minimizing the role of other muscles. The peroneus tertius is a comparatively small muscle, originating from the fibula and inserting on the fifth metatarsal base which gives it a different line of action than TA (Yammine and Erić, 2017). Hence its influence is negligible. Even though the physiological cross sectional area of EDL is 56% of TA (Wickiewicz et al., 1983) and its contribution to joint torque cannot be negligible, the EDL produces eversion with a line of action quite different from the TA (Kendall et al., 1993). A wedge was used to invert the ankle joint helped minimize the effects of EDL activity. Although EHL tendon does have a line of action positioned to produce inversion, it is at disadvantage both in terms of cross sectional area (only 18% of TA muscle; Wickiewicz et al., 1983) and mechanical attachment on the distal phalanx which requires toe and metatarsal extension before it can contribute to ankle dorsiflexion. To minimize its contribution, the participants were trained not to use their toes during voluntary contractions. This was observed throughout the experiments and trials were repeated whenever participants used other muscles.

TA muscle moment arm length with respect to ankle (talocrural joint) angle was previously reported to show inter-individual differences (Klein et al., 1996). Additionally, it does not change from 20 PF to 11 DF (see Figure 2A, Klein et al., 1996). Considering the ankle joint angles tested in the present study, the moment arm length was assumed to be constant and an increase in TA muscle length was anticipated from DF to PF positions. We found that ankle torque increased with increased TA muscle length imposed by a change in ankle angle from DF to Neutral and PF. This confirms the first part of the second hypothesis. Previous studies reported an increase of ankle dorsiflexion MVC torque from 10° DF to 10° PF position (Pasquet et al., 2005). Another earlier study reported max dorsiflexion torque at 10° PF (Marsh et al., 1981). These results were consistent also for the positions < 10° PF (van Schaik et al., 1994). Based on previous and present findings, we conclude that the optimum length of TA corresponds to a PF position.

Since significant correlations were found between IMP and ankle torque during ramp contractions, we expected that IMP would follow the torque-angle relationship that arises from mostly the TA muscle length-tension characteristics. However, the change in the magnitude of the IMP was not significant for different joint angles which rejected the second part of the second hypothesis. This indicates that there must be factors affecting the pressure other than muscle. One factor might be related to intramuscular alterations (Sejersted and Hargens, 1995), however, the location IMP sensor insertion was chosen to provide less variability. Previous modeling studies based on volumetric strain analysis using cine phase contrast MRI (Jensen et al., 2015) showed less regional volumetric strain in the superior and middle portion of TA for passive conditions (Jensen et al., 2015) and were used to guide sensor placement in this study.

The TA muscle fiber pennation angle has been shown to increase with contraction intensity (Maganaris, 2001). In contrast, it decreases with ankle position from dorsiflexion to plantar flexion (Maganaris, 2001). The amount of decrease is even more pronounced during MVC (Maganaris and Baltzopoulos, 1999; Pasquet et al., 2005). There is no previous data showing the relationship between IMP and pennation angle. However, it can be one of the determinants of IMP. This needs to be tested in future studies. If decrease in pennation angle causes drop in IMP, this may explain in part why the IMP at PF was not higher than the IMP at DF position. Other factors could be related to the compartmental pressure: (i) A recently developed two muscle model on the upper extremity, using the finite element method, showed that the pressure applied onto the bone substantially changes with respect to a change in elbow joint angle (Rohrle et al., 2017). This suggests that pressure on bone interacts with the mechanical characteristics of muscles that are located in a compartment and attached to the bone along their muscle length. (ii) Reinhardt et al. (2016) reported that intermuscular pressure between gastrocnemius medialis and plantaris muscles of NZ rabbits increased if the muscles are lengthened. Such *in situ* animal experiments comprising skin incisions or removals, and connective tissue dissections do not necessarily reflect *in vivo* conditions: e.g., fasciotomy has been reported to affect both intramuscular pressure (Garfin et al., 1981) and muscular forces (Ateş et al., 2013). Nevertheless, the findings of Reinhardt et al. indicate that intermuscular pressure changes with muscle length even after removing connective tissues. These recent modeling and experimental studies suggest that the increased pressure due to increased force production capacity of muscles can be shared with intercompartmental structures e.g., transferred elsewhere in the compartment either onto the bones or intermuscular structures. (iii) Intracompartmental and intercompartmental pressures were shown to be affected by joint position as well: Measured with slit catheters connected to a pressure transducer, the pressures of both anterior and deep posterior compartments were shown to be higher in the DF position (Tsintzas et al., 2009). In line with this, an earlier study using a similar method reported increased intracompartmental pressure for all lower leg compartments due to passive ankle DF (Gershuni et al., 1984). In summary, the IMP, which is not changing with the ankle joint angle, can be explained with the opposing effects of increased muscle force production against the decreased compartmental pressure at the PF position. However, this suggests that the characteristics of IMP is more complicated *in vivo* and it may not be easily interpreted to the conditions consisting of joint or muscle length changes.

The findings of this study confirmed our third hypothesis that the delay between the onsets of IMP and fwEMG activity is significantly shorter than the delay between the onsets of torque and fwEMG activity. The onset of sEMG activity is generally used for EMD calculations. In this study, EMD was computed based on the onset of fwEMG with the assumption that the local activity can be detected immediately by fine-wire electrodes. Therefore, it was expected that the time lag does not comprise the propagation time of the motor unit action potentials to the surface electrodes (Merletti et al., 2001). We found the EMD of the ankle torque to be higher than reported EMD for upper extremity muscles (Cavanagh and Komi, 1979; Norman and Komi, 1979), gastrocnemius (Hopkins et al., 2007), and close to the hamstrings (Blackburn et al., 2009), where EMD was calculated based on sEMG. These support the expectation. There are also other studies have reported high EMD values with respect to sEMG onset during high intensity voluntary contractions, e.g., 100–250 ms of EMD for quadriceps (Libardi et al., 2015). Hug et. al. recently argued that the EMD measurements performed during voluntary contractions are more prone to methodological artifacts. Due to the surface electrode location, error might be up to 20 ms (Hug et al., 2011). Onset delay calculations based on fwEMG eliminates this risk.

We observed negative onset delays for both IMP and sEMG. Fine-wire electrodes are collecting information from a limited area. Later onsets for fwEMG activity might be due to the activation threshold of the TA muscle fibers in the vicinity of the fine-wire electrodes. They may not be the first recruited fibers at the onset of the ramp contraction however; IMP sensor is still able to measure the pressure change. There is also a slight possibility that participants may move neighboring muscles without activating TA and the change in the position of other muscles might affect TA IMP. This may explain only the trials which show negative delay for IMP but not for sEMG. Even though it cannot be eliminated in any experimental settings that test voluntary contractions, this case existed very rarely (11 out of 126 trials) in the present experiments. EMD is determined by signal transduction e.g., propagation of the action potential and excitation-contraction coupling and transmission of muscle force through series elastic component comprised of contractile elements, aponeurosis, and tendon. By using non-invasive high frame rate ultrasound Nordez et al. (2009) showed the contribution of the passive part of the series elastic components of EMD for electrically stimulated gastrocnemius. An IMP sensor inserted close to the source of muscle activity can detect the passive series elastic component as well. More importantly, it gives the mechanical output simultaneously. Therefore, it is a promising minimally invasive method to detect impaired electromechanical transmission. Moreover, it is known that muscles may have heterogeneous onset of electrical activity (Holtermann et al., 2005). Recently, by using magnetic resonance and diffusion tensor imaging, heterogeneity of muscle fascicle strains were shown for gastrocnemius during low level of activity (Karakuzu et al., 2017). This indicates that local heterogeneities exist in active force production. With further testing, after improving the methodology e.g., using more than one sensor together with fwEMG, IMP can be used to detect both electrical and mechanical heterogeneities for high level muscle activation.

## Conclusions

Our study shows significant correlations between IMP, sEMG, fwEMG, and ankle torque during isometric ramp contractions consistent for all the ankle positions tested. This supports the idea that IMP captures mechanical properties of active muscle. While the ankle torque increases with the TA muscle length, the IMP does not follow the torque-ankle angle change. This is explained by the opposing effects of compartmental pressure that are higher at DF and with increased force at PF. Present findings suggest that the characteristics of IMP is more complicated *in vivo* and intramuscular mechanics may not represent the resultant effects measured on the joint. Additionally, the onset of IMP activity is found to be significantly earlier than the onset of torque which shows the potential of IMP method to detect muscular changes in the course of neuromuscular diseases.

## Author contributions

All authors contributed to the development of the project, experimental preparations, and data collection. FA: Performed data analysis and manuscript writing. KK: Contributed to writing, editing, and critical appraisal of the manuscript. All authors approved the final submitted manuscript.

### Conflict of interest statement

The authors declare that the research was conducted in the absence of any commercial or financial relationships that could be construed as a potential conflict of interest.
